# School children sufficiently apply life supporting first aid: a prospective investigation

**DOI:** 10.1186/cc7984

**Published:** 2009-07-31

**Authors:** Roman Fleischhackl, Alexander Nuernberger, Fritz Sterz, Christina Schoenberg, Tania Urso, Tanja Habart, Martina Mittlboeck, Nisha Chandra-Strobos

**Affiliations:** 1 Department of Emergency Medicine, Medical University of Vienna, Währinger Gürtel, Wien, 1090, Austria; 2 Core Unit for Medical Statistics and Informatics, Medical University of Vienna, Spitalgasse, Wien, 1090, Austria; 3 Division of Cardiology, Johns Hopkins Bayview Medical Center, Johns Hopkins University School of Medicine, 4940 Eastern A, Baltimore, 21224-2780, Maryland, USA

## Abstract

**Introduction:**

The usefulness of CPR training in schools has been questioned because young students may not have the physical and cognitive skills needed to correctly perform such complex tasks correctly.

**Methods:**

In pupils, who received six hours of CPR training from their teachers during a standard school semester at four months post training the following outcome parameters were assessed: CPR effectiveness, AED deployment, accuracy in checking vital signs, correctness of recovery position, and whether the ambulance service was effectively notified. Possible correlations of age, gender, body mass index (BMI), and outcome parameters were calculated.

**Results:**

Of 147 students (mean age 13 ± 2 years), 86% performed CPR correctly. Median depth of chest compressions was 35 mm (inter quartile range (IQR) 31 to 41), and the median number of compressions per minute was 129 bpm (IQR 108 to 143). Sixty nine percent of the students tilted the mannequin head sufficiently for mouth to mouth resuscitation, and the median air volume delivered was 540 ml (IQR 0 to 750). Scores on other life supporting techniques were at least 80% or higher. Depth of chest compressions showed a correlation with BMI (r = 0.35; *P *< 0.0001), body weight (r = 0.38; *P *< 0.0001), and body height (r = 0.31; *P *= 0.0002) but not with age. All other outcomes were found to be unrelated to gender, age, or BMI.

**Conclusions:**

Students as young as 9 years are able to successfully and effectively learn basic life support skills including AED deployment, correct recovery position and emergency calling. As in adults, physical strength may limit depth of chest compressions and ventilation volumes but skill retention is good.

## Introduction

Prompt Basic Life Support (BLS) improves survival in patients after cardiac arrest [1]. As a consequence many agencies have targeted efforts at training lay people in cardiopulmonary resuscitation (CPR) skills. Over the past eight years, this curriculum has been simplified to improve retention and increase its appeal to the lay rescuer. Three-hour BLS training programs have evolved to 30 minute programs [2-4]. Driven largely by the understanding that the knowledge of CPR represents a core skill, some investigators have reported on the success of teaching simplified BLS skills to 11 year old school children [5]. These studies, albeit encouraging, represented only preliminary observations and did not evaluate varying age groups of students nor did they address the various parameters or predictors of good CPR performance by such young persons. Such studies would be essential if the goal was to develop effective school-based training programs for students [6]. Therefore, several educationists have questioned the validity of implementing such a wide-spread curriculum because the parameters to guide implementation have been poorly addressed, that is when does a school child have the physical and/or cognitive ability to learn and implement CPR? [7] Can the potential life-saving techniques, such as placing an emergency call, providing CPR or using an automatic external defibrillators (AED), appropriately be taught to young students? The goal of this study was to answer these questions – even if restricted by practical reasons to children aged from 9 to 18 years – and thereby better guide National Education Policy and its implementation.

## Materials and methods

This study was designed as a prospective investigation in volunteer schools, both urban and rural, scattered across Austria. The study was approved by the Ethics Committee of the Austrian Red Cross, Vienna branch. Eleven randomly selected schools in four states were recruited and required to teach students ranging in age from 9 to 18 years. The teachers, who would train the students, were all faculty at their individual schools and volunteered to participate. All were trained by the Austrian Youth Red Cross to the level of a BLS instructor using a standardized curriculum.

### Curriculum content

Students were instructed life-supporting skills according to an implemented standard curriculum for approximately six hours as shown in Table 1. Skills taught included using an AED, providing CPR, and treating life-threatening bleeding, with the course comprising didactic sessions plus hands on training on mannequins. Classes spanned a time period of approximately three months.

**Table 1:** Performance checklists

Emergency call	
	✓ Correct telephone number of ambulance service?
	✓ Address given
	✓ Situation/accident/illness explained
	✓ Number of victims given
	✓ Call back telephone number provided
Check for vital signs	
	✓ Responsiveness checked
	✓ Gently shook patient
	✓ Called for help
	✓ Opened airways correctly
	✓ Checked for normal breathing

Recovery position	
	✓ Positioned volunteer in stable recovery position
	✓ Opened airway sufficiently
	✓ Directed mouth downwards

### Investigation protocol and student identification

In 11 volunteer schools across Austria, 180 students were trained in CPR between 9 May and 2 June 2006. Students ranged in age from 9 to 18 years and were usually in grade 4 to the final year of high school. At the end of the school year, investigators visited the schools to conduct a standardized evaluation of skills learned. To avoid selection bias, whole school classes were taught and invited to join evaluation. The class selection was simply given through the volunteering teacher and her or his allocation to a particular class. Anyhow, students were given the opportunity to withdraw from study participation.

The parents of all students had been informed by the local teachers and asked to give their informed consent for their children to join our evaluation. Parents who gave consent were then asked to provide weight and height measurements of their children. Prior to evaluation, the children were asked to give their consent to participate in our investigation. The evaluations were conducted in a private room, separate from where the other students and teachers waited. Parents were allowed to be present during the evaluation, if they or their children wanted.

For automated data collection we used a Resusci-Anne™ Skill Meter (Laerdal Medical AS, Stavanger, Norway). All evaluations were carried out by investigators who had not participated in training the teachers or the students.

### Measurements

We documented age, gender, body weight, and height of all participants. These data were used to calculate the body mass index (BMI) of each participant. To evaluate CPR performance, the training mannequin recorded various effectiveness parameters, such as the ratio of ventilation to chest compressions, depth of chest compressions, frequency of compressions, correct head tilt and chin lift, and correct breathing volumes. The time needed to administer an effective electric shock by using an AED was measured with a stop watch. For evaluation we used the same AED models that the students had used during training (CRT^©^, Medtronic, Minneapolis, MN, USA; Heartstart^©^, Heartstream-Philips, Seattle, WA, USA; Fred easy^© ^Trainer, Schiller Medical, Switzerland). AED deployment was considered correct if the two pads were unpacked, removed from the plastic liners, and attached to the bare chest of the mannequin according to current European Resuscitation Council (ERC) recommendations [8]. The shock button had to be pressed after the appropriate voice prompt was given by the device. The stop watch was started after the investigator gave a verbal command to start deploying the AED, without a check of vital signs.

The simulated phone call, the check for vital signs, and the ability to place a volunteer in a stable recovery position were scored by an investigator according to standardized checklists shown in Table 1. The checklists were developed according to the ERC guidelines for adult BLS and the curriculum for first aid training of the Austrian Red Cross [8,9] and are commonly used for skills assessments.

### Outcomes

The primary outcome evaluated was CPR effectiveness defined as the ratio of chest compressions to ventilations, chest compression depth in mm, the number of chest compressions per minute (bpm), sufficient head tilt and chin lift during artificial breathing, volume in ml inflated during mouth to mouth resuscitation, correct usage of an AED, and time until successful AED deployment in seconds.

For secondary outcomes, we calculated the rate of correct emergency calls, adherence to guidelines during the check of vital signs, and the ability to establish a correct recovery position (Table 1).

### Statistics

This prospective, single-arm intervention study was designed to investigate associations between demographic data and CPR outcomes. Categorical data are described by absolute numbers and percentages. Continuous data were described by medians and interquartile ranges (IQR), because most of our data did not show a normal distribution. Correlations between continuous data were assessed using the non-parametric Spearman rank correlation coefficient, and differences between groups were tested by the Wilcoxon rank-sum test. To avoid the loss of details on chest compression depth or tidal volume, we decided not to dichotomise data. Regression models were used to simultaneously assess the effect of age, BMI, and gender on predefined outcomes. In this case, appropriate transformations were performed so that all residuals had equal variability and so that residuals showed an approximately normal distribution. All *P *values are two-sided and a *P *< 0.05 was considered to be statistically significant.

## Results

In this study 180 students were trained in their respective schools. Of these students 16 (8.9%) refused testing, one (0.6%) had a scheduling issue, and one (0.6%) agreed only to the evaluation of the emergency call and recovery position. Thus, 162 students underwent evaluation, 151 were present on the day of practical testing and finished all parts of the evaluation. None of the students had attended first aid training before participating in this study. The average time from the last class to the evaluation session was 120 days. Demographic data are displayed in Table 2. Students tested included those with special learning needs; most students were age appropriate for their education grade class (e.g. 9 years of age in the fourth grade of primary school).

**Table 2:** Demographics

	25%	50%(Median)	75%	Min	Max
	(boys/girls)	(boys/girls)	(boys/girls)	(boys/girls)	(boys/girls)
Bodyweight (kg)	40 (42/40)	50 (56/48)	60 (65/52)	27 (28/27)	120 (120/72)
Height (cm)	150 (150/150)	160 (168/160)	173 (180/164)	132 (133/132)	188 (188/184)
BMI	17.6 (18.4/16.8)	19.2 (19.8/18.5)	20.9 (21.3/20.3)	13.9 (15.1/13.9)	37.0 (37.0/27.4)
Age (years)	11 (12/11)	14 (14/13)	15 (16/15)	9 (9/9)	18 (18/17)

Data are displayed as absolute numbers, medians, and interquartile ranges. Data are shown in total and in parenthesis for boys and girls separately (n = 162).

BMI = body mass index.

### Basic life support skills

Students (n = 151) were tested for their knowledge of the telephone number of the local emergency medical services and 95% gave it correctly. The correct address and nature of the emergency was provided by 98%, the adequate number of victims indicated by 96%, and their contact details for possible call back was left by 93%.

These 151 students were also tested for their adherence to a checklist during the check for vital signs; 85% assessed responsiveness, 83% gently shook the patient, and 44% called for help. The airway was opened correctly by 70% and 80% checked for breathing.

Due to technical problems during CPR evaluation, some subsets of data (e.g. ventilation) were recorded incorrectly by a few students. Thus data for 147 students is reported for students demonstrating CPR on an evaluation mannequin. In this group, 86% performed CPR technically correctly, providing 30 chest compressions, followed by two breathes. However, only 69% of all students tilted the head to open the airway for mouth-to-mouth ventilation (Table 3).

**Table 3:** Primary outcome variables

Effectiveness parameter	Median	IQR	Min	Max
Depth of chest compressions*	35 mm	31 to 41 mm	8 mm	56 mm
Number of chest compressions per minute	129 bpm	108 to 143 bpm	51 bpm	203 bpm
Volume inflated during mouth-to-mouth resuscitation	540 ml	0 to 750 ml	0 ml	1790 ml
Time until AED shock	69 s	55 to 84 s	25 s	150 s

	**Correct**	**%**		

Correct head tilt and chin lift during artificial ventilation	102	69%		
Number of students who correctly deployed the AED	137	93%		

*Presented as medians and inter-quartile ranges (IQR), even if the data were nearly normally distributed. Data are displayed as absolute numbers or percentages of correctly shown checklist items. In absence of a normal distribution, median, interquartile range, and range are given (n = 147).

AED = automatic external defibrillators.

Of the 151 data sets analyzed for correct placement in the recovery position, 97% of the students were successful, and, in addition, 68% tilted the head back to open the airway.

### Determinants of appropriate chest compression

**Figure 1:**
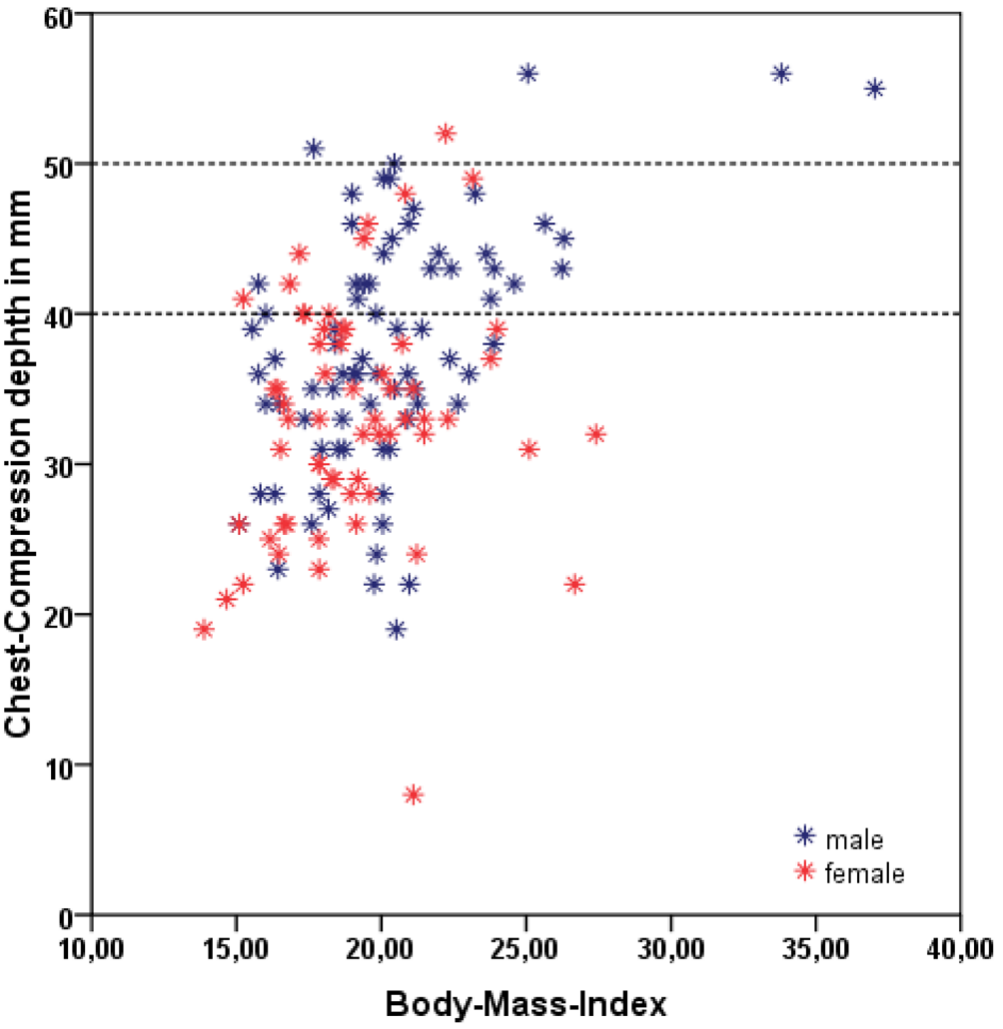
Scatter plot demonstrating the relationship between depth of chest compressions in mm and body mass index. The recommended chest compression depth of 40 to 50 mm is marked by two dotted lines.

**Figure 2:**
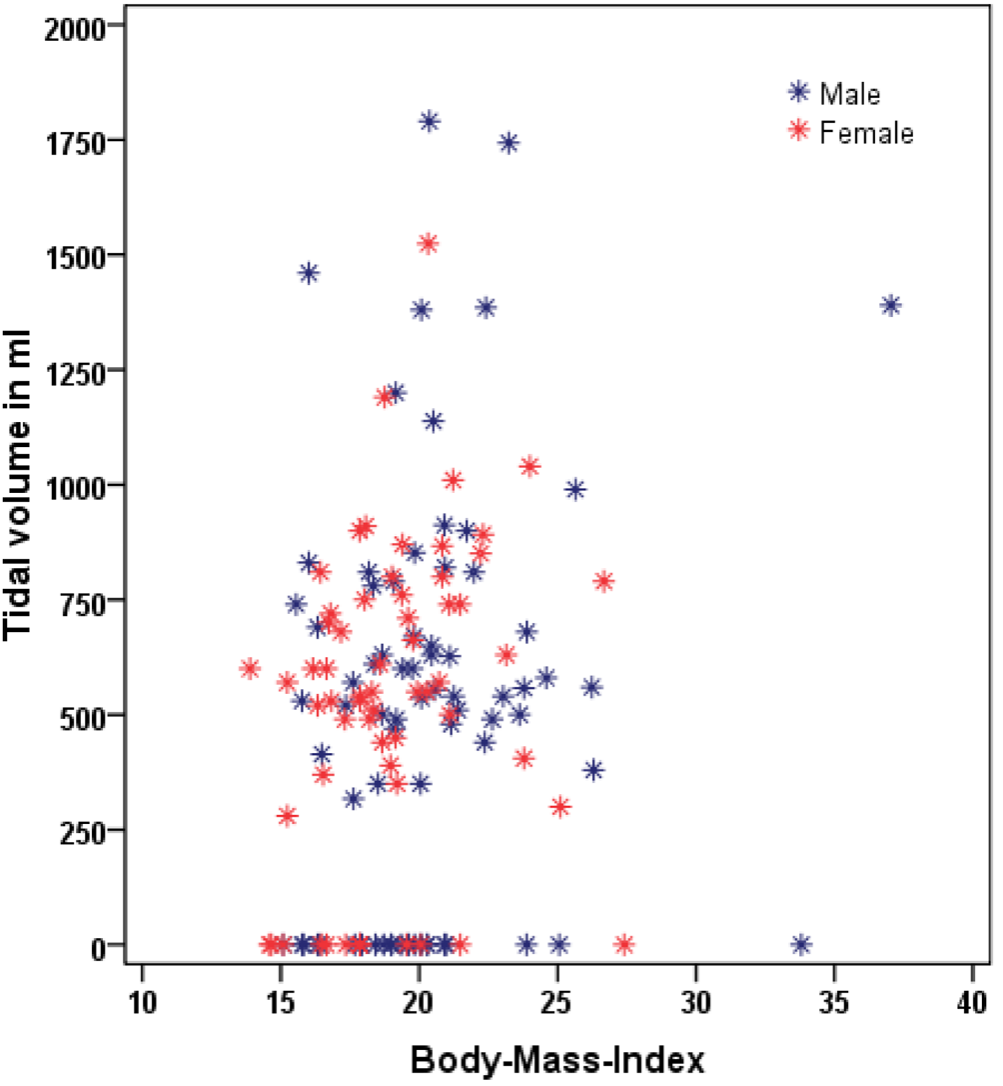
Scatter plot demonstrating the relationship between tidal volume (ml) and body mass index.

The influence of age on the depth of chest compression was not significant (r = 0.14; *P *= 0.10). In contrast, the depth of compression was dependent on BMI (r = 0.35; *P *< 0.0001), and individually on body weight (r = 0.38; *P *< 0.0001), and body height (r = 0.31; *P *= 0.0002). Furthermore boys performed deeper chest compressions than girls, with the difference being statistically significant: the boys compressed to a median depth of 37 mm (IQR 33 to 43), whereas the girls compressed to a median depth of 33 mm (IQR 28 to 38, *P *= 0.0015). Correlation of BMI with the depth of chest compression or with the volume of air administered during mouth-to-mouth resuscitation is depicted in scatter plots in Figures 1 and 2.

When we considered a multivariate model with age, BMI, and gender as independent covariates, BMI (*P *< 0.001) and gender (*P *= 0.016) were found to be significant as independent prognostic covariates. At a BMI of 15 or greater adequate chest compression could be attained.

### Determinants of appropriate ventilation

The volume inflated during artificial breathing was not significantly affected by body height (r = 0.14; *P *= 0.10), age (r = 0.16; *P *= 0.06), or gender (*P *= 0.70). However, body weight (r = 0.18; *P *= 0.04), and, by extension, BMI (r = 0.21; *P *= 0.01) were statistically significant factors in determining tidal volume delivered during mouth-to-mouth resuscitation.

In terms of airway opening and correctly doing a head tilt, these skills were not dependent on BMI or gender (*P *= 0.05): 25 boys incorrectly tilted the head and lifted the chin, compared with 52 who did this correctly; the corresponding numbers for girls were 13 and 49 (*P *= 0.13). The median age for students who incorrectly tilted the head was 12 years (IQR 11 to 15), compared with a median age of 14 years (IQR 12 to 16) for those who did it correctly (*P *= 0.08).

We further analyzed those 52 students who sufficiently ventilated the resuscitation mannequin, in order to understand more clearly the relation of tidal volume, BMI, age and gender. The median tidal volume supplied by these students was 619 ml and ranged from 317 to 1790 ml (IQR 515 to 825 ml; n = 52). Neither gender (*P *= 0.7) nor age (*P *= 0.44) influenced tidal volume during mouth-to-mouth resuscitation. BMI, on the other hand, again showed a statistically significant correlation with tidal volume (*P *= 0.03). With BMI of 14 or greater, adequate ventilation volumes were generally attained.

Table 4 lists the various skills taught, the percentage rate of success in learning these skills, and the mean age and BMI of students performing these skills successfully and those who were unsuccessful.

**Table 4:** Percent of success in learning the skills taught

	Number	Percentage of success	Mean age	Mean BMI	Percentage of failure	Mean age	Mean BMI
**Call ambulance**	151	95%	13.5	19.2	5%	12.8	20.1
**Check vitals signs**	151	85%	13.7	19.6	15%	12.9	18.4
**Recovery position, head tilt**	151	70%	13.6	19.1	30%	13.6	20
**AED**	147	93%	13.6	19.4	7%	12.9	17.6

The number of students tested for the specific category as listed, the % of students who performed the task correctly, the mean age and the mean body mass index (BMI) of students performing the task correctly in relation to those who did not.

AED = automatic external defibrillators.

## Discussion

The overwhelming majority of students, ranging in age from 9 to 18 years had no difficulties retaining standard CPR techniques after they had been trained by their teachers for approximately six hours.

Age did not influence the depth of chest compressions or tidal volume mouth-to-mouth resuscitation, suggesting that children as young as nine years old could effectively learn such critical skills. Depth of chest compressions or tidal volume during mouth-to-mouth ventilation, were influenced most by the BMI of the student (r = 0.21; *P *= 0.01). However, body weight (r = 0.18; *P *= 0.04) on the one side, and age (r = 0.16; *P *= 0.06) on the other are in close proximity to the significance cut-off at *P *< 0.05 and should not lead to any kind of dichotomous thinking that would be potentially misleading.

Our results are in keeping with other similar observations that have been forthcoming from the adult learner literature [10,11]. BMI did not influence cognitive or technical skills such as performing a correct emergency call, establishing recovery position, deploying the AED correctly, or performing CPR with the correct ratio of breathing to chest compressions. Students tended to give chest compressions that were slightly too frequent. Studies with adult populations show a similar tendency [10]. It is compelling to note that the retention and performance of these young students is remarkably similar, if not better, than that reported in adults [10]. In light of the 31% failure to tilt the head back during artificial ventilation, our investigation also demonstrates the fact that this skill is complex and likely requires greater training and practice, different teaching methods or maybe just a more positive but strict feedback from trainers. However, clarification was beyond the scope of this investigation.

Given the excellent performance by the students evaluated in this study, the data support the concept that CPR training can be taught and learnt by school children and that CPR education can be implemented effectively in primary schools at all levels. Even if physical strength may limit CPR effectiveness, cognitive skills are not dependent on age, and with periodic retraining, children's performance would likely improve over time [12]. Although the median depth of chest compression achieved by very young children (aged nine years) was generally too shallow for adult BLS based on ERC recommendations, it did achieve the recommended depth for resuscitation of children [13] suggesting that at the very least, children can help others of their age and also learn skills vital to improving the chain of survival, i.e. early notification of emergency medical services systems [14].

Many educational institutions hesitate to include first aid training in the scholastic curriculum [15]. With the available literature pointing to hesitation by adults to perform first aid [16], and the poor performance of life-saving measures [17], including CPR training at young ages in schools could be an effective solution, to improve bystander initiated rescue efforts.

It is important to recognize that this study had no control group to assess the actual change in skill or knowledge as measured before the training program was instituted. We note that most studies of teaching CPR skills have never employed a control group. The premise is fundamentally that the control group may have an intrinsic amount of basic CPR knowledge but clearly it is inadequate. We freely acknowledge that some basis knowledge may exist (such as calling an emergency number), while other aspects of the skills taught are unlikely to exist in the knowledge base of the individual being taught. Also the study was not randomized for the same purpose. The fundamental premise remains that the reason why BLS is taught to the public is that there is a presumption that the knowledge base does not exist in the population.

This study is limited in that it did not study students younger than nine years of age. Previous studies showed good skill acquisition and retention in students aged 8 to 11 years after they had attended specialised 'under 11 rescuer' first aid training [5]. Furthermore, we had previously carried out similar studies on children six to seven years old, and we had found that they performed well when calling emergency medical services or establishing the recovery position. However, CPR skills showed a median score of 3.5 (95% confidence interval = 1.5 to 3.6) on a six-item scale (from 1 'excellent' to 6 'insufficient') [18].

This study was performed in 2006 and American Heart Association and ERC guidelines at that time encompassed the training of ventilation during BLS. Since then studies have suggested the lesser importance of teaching ventilation to lay people and new guidelines for lay person BLS have been proposed reducing the need to train lay people in ventilation. Our study demonstrates, yet again, that ventilation is a difficult skill to be taught and retained.

## Conclusions

Our data demonstrate that standard CPR training can be effective learnt by school children above the age of nine years. Skills such as calling emergency medical services, deploying an AED, or placing the victim in the recovery position can be effectively performed by school children after only six hours of effective instruction and practice. For at least the 120 days studied, the retention of these skills is good if not better that that of adult learners. Young age does not limit the learning of CPR cognitive skills, but lack of physical strength may. The advantages of early activation of the emergency medical services and constant retraining is likely to outweigh the limitations of physical strength. Training can be provided by the students' own teachers, if they have been appropriately trained themselves.

This paper demonstrates the parameters based on which resuscitation skills can be taught in a school. Together with existing literature for the ages of six to eight years it defines minimal age, and more importantly describes that learning and providing CPR skills is related to physical ability rather than chronological age. It clearly has implications on how and who will be taught in school. It strongly endorses the fact that these skills can be taught and retained for at least 120 days. It lends further weight to the recommendation that has come from many agencies that this skill can and should be taught in schools. If the goal of primary school training is to teach the basic skills of education and survival, that is reading, writing and arithmetic (the 3 Rs), perhaps as educators we need to take one giant step forward and introduce the fourth R – Resuscitation.

## Key messages

• Pupils received six hours of CPR training from their teachers during a standard school semester.

• CPR effectiveness, AED deployment, accuracy in checking vital signs, correctness of recovery position, and whether the ambulance service was effectively notified can be taught in a school.

• Skill retention is good and related to physical ability rather than chronological age.

• Students as young as nine years old are able to successfully and effectively learn BLS skills.

## Abbreviations

AED: automatic external defibrillators; BLS: basic life support; BMI: body mass index; CPR: cardiopulmonary resuscitation; ERC: European Resuscitation Council; IQR: inter quartile range.

## Competing interests

The authors declare that they have no competing interests.

## Authors' contributions

RF carried out conception and design of the study, acquisition, analysis and interpretation of data, drafting of the manuscript, critical revision of the manuscript for important intellectual content and administrative, technical, and material support, such as supervision of the study. AN, CS, TU, and TH participated in acquisition of data, critical revision of the manuscript for important intellectual content and carried out administrative, technical, and material support. FS participated in conception and design of the study, acquisition, analysis and interpretation of data and carried out critical revision of the manuscript for important intellectual content, administrative, technical, and material support, such as supervision. MM participated in conception and design of the study, analysis and interpretation of data, and critical revision of the manuscript for important intellectual, carried out statistical analysis and administrative support. NC participated in conception and design of the study, analysis and interpretation of data, and carried out critical revision of the manuscript for important intellectual content. All authors read and approved the final manuscript.
